# Atorvastatin (Lipitor) attenuates the effects of aspirin on pancreatic cancerogenesis and the chemotherapeutic efficacy of gemcitabine on pancreatic cancer by promoting M2 polarized tumor associated macrophages

**DOI:** 10.1186/s13046-016-0304-4

**Published:** 2016-02-16

**Authors:** Qiaofei Liu, Yuan Li, Zheyu Niu, Yi Zong, Mengyi Wang, Lutian Yao, Zhaohui Lu, Quan Liao, Yupei Zhao

**Affiliations:** Department of General Surgery, Peking Union Medical College Hospital, Chinese Academy of Medical Sciences and Peking Union Medical College, 1# Shuai Fu Yuan, Dong Dan District, Beijing, 100730 China; Department of Pathology, Peking Union Medical College Hospital, Chinese Academy of Medical Sciences and Peking Union Medical College, Beijing, 100730 China

**Keywords:** Pancreatic cancer, Cancerogenesis, Chemotherapy, Gemcitabine, Aspirin, Atorvastatin, Myeloid derived suppressor cells, Tumor associated macrophages

## Abstract

**Background:**

Interactions of inflammatory cells with pancreatic cancer cells play crucial roles in pancreatic cancer, however the dynamic changes of inflammatory cell populations in pancreatic cancerogensis and after chemotherapy have not been well eclucidated. The combinational use of aspirin and atrovastatin (Lipitor) have been widely prescribled for cardio-cerebral vascular diseases mainly by regulation of inflammations, and they have been also reported to have plausible anti-tumor effects, however their potential roles in pancreatic cancerogenesis and chemotherapeutic effects have been seldom investigated. We scanned the dynamic changes of pan-inflammatory cell populations in pancreatic cancerogensis and after chemotherapy and found the potential target cell populations. Then we tested the roles of aspirin and Lipitor to regulate these inflammatory cell populations and their effects on pancreatic cancerogenesis and chemotherapeutic effects.

**Methods:**

Cancerogen, dimethylbenzanthracene (DMBA), was used to induce pancreatic cancerogenesis and subcatunous implantation of syngenic murine Panc02 pancreatic cancer cells was adopted as well. Gemcitabine was used for chemotherapy. The peripheral blood, pancreatic lesions and tumor samples were harvested and analyzed to search for the potential target cell populations. The roles of aspirin and Lipitor to regulate these cell populations and their potential effects on pancreatic cancerogenesis and chemotherapeutic efficacy were investigated both in vitro and in vivo.

**Results:**

We found progressive accumulations of myeloid-derived suppressor cells (MDSC) and M2-polarzied tumor associated macrophages(M2) in pancreatic lesions accompanied with dynamic reducations of cytotoxic T cells(CTL) and helper T cells(Th) in the progression of pancreatic cancerogenesis. After gemcitabine treatment, the MDSC significantly reduced, however M2 soared up unexpectedly. Aspirin could significantly inhibit the MDSC and M2 to prevent pancreatic cancerogenesis and improve chemotherapeutic effects of gemcitabine, however Lipitor did not significantly affect MDSC, instead it could promote M2 to attenuate the postive effects of aspirin and gemcitabine.

**Conclusions:**

MDSC and M2 accumulate in progression of pancreatic cancerogenesis and gemcitabine can induce M2. Aspirin could prevent pancreatic cancerogenesis and improve efficacy of gemcitabine partially by inhibiting MDSC and M2, however when used in combination, Lipitor could weaken the efficacy of aspirin and gemcitabine partially by promoting M2.

**Electronic supplementary material:**

The online version of this article (doi:10.1186/s13046-016-0304-4) contains supplementary material, which is available to authorized users.

## Background

Pancreatic cancer is a devastating disease with a rising incidence, rendering it the fourth most common cause of cancer-related mortality [1]. Pancreatic cancer proceeds through a morphological spectrum of tumor precursor lesions, named pancreatic intradutal neoplasia (PanIN), which makes PanINs as potential targets for the prevention of pancreatic cancer [2]. Chemotherapy is the main treatment for unresectable pancreatic cancer patients. Gemcitabine is used as a first-line drug, however, the objective response rate to gemcitabine is approximately 10–20 % and the secendary drug resistance is high up to 80 % [3]. The pathological traits of pancreatic cancer are exclusive with highly dense fibrosis and abundant infiltration of inflammatory cells which have been reported to affect pancreatic cancerogenesis and the chemotherapeutic effects of gemcitabine [4]. Although myeloid derived suppressor cells (MDSC) [5], tumor associated macrophages (TAM) [6] and dendritic cells (DC) [7] have been reported to affect the biological behaviors of pancreatic cells and the efficacy of chemotherapy and immunochemotherapy, the dynamic changes of pan-inflammatory cell populations in the process of pancreatic cancerogenesis and after chemotherapy are little known. So systematic scanning of the dynamic changes of pan-inflammatory cell populations in the process of pancreatic cancerogenesis and after chemotherapy can provide important information for further understanding the interactions of inflammatory cells with pancreatic cancer and chemotherapy which can be of great value to prevent pancreatic cancer and improve the chemotherapeutic efficacy.

Aspirin has strong effects to inhibit inflammations and it have been also reproted to inhibit the immunosuppressive MDSC [8]. Atorvastatin (Lipitor), one of the statins, always prescribled with aspirin to prevent and treat cardo-cerebral vascular disease by lowering down sera lipid and it was also reported that Lipitor could inhibit the activation of macropahges [9, 10]. Recent years, these two drugs have been reported to have plausible anti-tumor effects, however the results were conflicting [11–13], and their effects on pancreatic cancerogenesis and chemotherapy were seldom investigated. Considering the potential roles of these two drugs in regulation of cancer related inflammations, we initially hypothesized that aspirin and Lipitor could probably have preventive roles for pancreatic cancer and improve the chemotherapeutic efficacy of gemcitabine.

Herein, after scanning 11 inflammatory cell populations in the process of pancreatic cancerogenesis and after chemotherapy, we found MDSC and M2-polarized TAM(M2) changed most significantly which could probably be powerful targets for prevention of pancreatic cancerogenesis and improving chemotherapeutic efficacy. Consquently, aspirin could inhibit the expansion of MDSC and M2, however, Lipitor promted M2. Aspirin substantially prevented the pancreatic cancerogenesis and improved chemotherapeutic effects of gemcitabine partially by inhibiting MDSC and M2, however Lipitor weakened the positive roles of aspirin and gemcitabine by promoting M2.

## Methods

### Mice

Male 4-week-old or 8-week-old C57BL/6J mice were purchased from VITAL RIVER Animal Center (Beijing, China). All of the animals were maintained in a pathogen-free animal facility for at least 1 week prior to use. Animal studies were performed in accordance with the institutional guidelines of Peking Union Medical College Hospital and were approved by the Lab Animal Committee of Peking Union Medical College Hospital.

### Reagents and antibodies

Gemcitabine was purchased from Eli Lilly (Indiana, USA). Granulocyte–macrophage colony-stimulating factor (GM-CSF) were purchased from BD Biosciences (New Jersey, USA). Aspirin, atorvastatine (Lipitor), carboxyfluorescein succinimidyl amino ester (CFSE), dimethylbenzanthracene (DMBA) and dimethyl sulfoxide (DMSO) were purchased from Sigma-Aldrich (St. Louis, USA). CV-7 PTFe suture lines were purchased from GORE-TEX Suture (W.L. Gore & Associates, Inc, Arizona, USA). The antibodies were purchased from BD Biosciences (New Jersey, USA)., Biolegend (San Diego, USA) or Santa Cruz Biotechnology (Texas, USA). All of the antibodies used in this study were listed in the (Additional file 1: Table S1).

### Cell lines and bone marrow cells

The murine Panc02 pancreatic cancer cell line is a sarcoma-like adenocarcinoma cell line that was derived from a 3-methylcholanthrene-induced tumor in a C57BL/6J female mouse. The murine monocyte cell line RAW 264.7 was purchased from Cell Line Center of Chinese Academy of Medicine Sciences. These two cell lines were maintained in high glucose DMEM (Dulbecco’s modified Eagle medium; Gibco BRL Co. Ltd., USA) supplemented with 10 % FCS (fetal calf serum; Gibco BRL Co. Ltd., USA) and 1 % penicillin and streptomycin. Bone marrow cells (BMCs) from the femurs of 4-week-old C57BL/6J mice were prepared as previously described [14]. When preparing BMCs, the T and B lymphocytes in bone marrow were removed using CD3^−^T cell and CD19^−^B cell positive selected magnetic beads (MACS, MiltenyiBiotec., BergischGladbach, Germany) according to the manufacturer’s protocols.

### Flow cytometery

The peripheral blood was lysed using a red blood cell lysing buffer according to the manufacturer’s protocols (Red Blood Cell Lysing Buffer Hybri-Max™; Sigma-Aldrich, USA). The subcutaneous tumor samples or the pancaretic lesions were harvested by blunt dissection. These tissue samples were cut into small pieces and minced using scalpel blades. Then, the minced pieces were mixed with 200 U/mL collagenase IV (Sigma-Aldrich, USA) and incubated at 37 °C for 1.5 h for enzymatic dissociation. At the end of the incubation, the cells were filtered through a 70-μm filter. The single cell suspension was collected in ice-cold phosphate buffered saline (PBS) and incubated with the antibodies in the darkroom for 30 min. Next, the stained cells were resuspended in PBS with 1 % paraformaldehyde and stored at 4 °C prior to flow cytometric analysis (Accuri C6, BD, USA). For each analysis, an isotype-matched monoclonal antibody was used as a negative control. The total inflammatory cells (CD45^+^), total T lymphocytes (CD3^+^), T helper cells (Th) (CD3^+^ CD4^+^), cytotoxic T lymphocytes (CTL) (CD3^+^ CD8^+^), B lymphocytes (CD19^+^), granulocytes (Gr-1^+^), dendritic cells (CD11c^+^), NK cells (NKP46^+^), NKT cells (CD3^+^NK1.1 ^+^), monocytes & macrophages (F4/80^+^), M1-polarized tumor associated macrophages (TAM) (F4/80^+^CD16/32^+^CD206^−^), M2-polarized TAM (F4/80^+^CD16/32^+^CD206^+^) and myeloid derived suppressor cells (MDSC) (CD11b^+^Gr-1^+^) were detected. Each experiment was carried out in triplicates.

### Hematoxylin-eosin staining

The tumor tissue and pancreatic lesion samples were fixed in 10 % phosphate-buffered polyformalin and embedded in paraffin. The formalin-fixed and paraffin-embedded samples were cut into 4-μm thick sections, mounted on poly-L-lysine–coated slides (Sigma-Aldrich, St. Louis, MO), and dried over night at 37 °C. The sections were then dewaxed in xylene and rehydrated according to the standard histopathological procedures and stained with hematoxylin and eosin.

### Real time reverse transcribed polymerase chain reaction (Real time RT-PCR)

The sorted cells, spleens and the tissues (fresh or frozen) without obvious necrosis was homogenized and lysed with Trizol reagent (Life Technologies, USA), and the total RNA was extracted using an RNeasy kit (Qiagen, Hilden, Germany) according to the manufacturer’s protocol. The total RNA was reverse transcribed into cDNA using a high capacity RNA-to-cDNA kit (Life technologies, USA). All the PCR reactions were carried out on a Bio-Rad CFX96 PCR System (USA). The first step was performed at 95 °C for 10 min, and the mRNA expression levels were determined over 35–40 cycles with 15 s of denaturation at 95 °C and annealing-extension-data acquisition at 52–60 °C for 60 s using a Power SYBR®Green PCR kit (UltraSYBR Mixture, KAPA SYBR® FAST qPCR Kits). All the primer sequences are listed in the (Additional file 1: Table S2). The relative fold mRNA expression levels were determined using the comparative Ct (ΔΔCt) method. All the reactions were carried out in triplicates.

### Elisa test

The sorted cells, spleen, and the fresh or frozen tumor tissues were harvasted and lysed for elisa test. All of the commercial sandwich ELISA kits used for the quantitation of IL-2, IL-4, IL-6, IL-10, IL-12, IFN-γ, TNF-α and TGF-β were purchased from R&D systems Inc.. The OD value of each of the samples was measured at 450 nm using a SpectraMax190 ELISA plate reader. Cytokine levels were quantified from three titrations using standard curves and expressed in pg/ml.

### Western blotting

Cultured cell samples were immersed in protein extraction buffer and centrifuged. The supernatant was removed and the protein concentration was measured using the BCA method (Pierce, MO, USA). Twenty micrograms of total protein was prepared for electrophoresis through an 8 % (v/v) sodiumdodecyl sulfate/polyacrylamide gel and transferred to a polyvinyli-denefluoride (PVDF) membrane. After incubation in 5 % (w/v) nonfat dry milk for 0.5 h at room temperature to block non-specific binding, the PVDF membrane was incubated with primary antibodies overnight with agitation at 4 °C. The membrane was then washed three times for 5 min each in TBST and incubated with HRP-conjugated secondary antibodies (1:2000; Golden Bridge Biological Technology, Beijing, China) in 5 % (w/v) nonfat dry milk and TBST for 30 min at room temperature with agitation. After washing three times in TBST, the bands were visualized with an Enhanced Chemiluminescence system (Millipore, Billerica, USA). The arginase-1 and CD206 antibodies (Santa Cruz, USA) were used, respectively.

### Myeloid dervied suppressor cells (MDSC) and tumor asssociated macrophages (TAM) indcued by pancreatic cancer in vitro

The tumor supernatant from Panc02 cells were collected when 90 % of the confluence of Panc02 cells was achieved in culture dish. The conditional medium was perpared by mixing 80 % of the high glucose DMEM containing 12.5 % FCS with 20 % of the tumor supernatant. The conditional medium was used to induce MDSC and TAM in vitro. To induce MDSC and M2, 10 ng/ml of GM-CSF was added into the conditional medium as well. The cells were harvested 72 h later. For analyzing the roles of different drugs, 18 h after incubation, the cells were divided into 4 groups, respectively: the control group, the aspirin group (ASP, 5 mmol/l), the Lipitor group (LIP, 40 μmol/l), and the aspirin + Lipitor group (ASP + LIP, aspirin 5 mmol/l, Lipitor, 40 μmol/l). The aspirin was first dissolved in dimethyl sulfoxide (DMSO) and then added to DMEM, with a final DMSO concentration of less than 1 %. The cells were harvested 72 h.

### Murine pancreatic cancerogenesis model

To establish the spontaneous pancreatic cancerogenesis model, the DMBA implantation in the distal pancreas method was applied. Briefly, the mice were submitted to pre-operative fasting for 6 h. The mice were anesthetized with a chloral hydrate solution i.p injection (10 %, 20 μl/10 g). A median laparotomy was performed; the incision was approximately 1 cm long, and the distal pancreas and spleen were gently removed from the abdominal cavity. A purse-string suture of 10 mm in diameter was performed using 7–0 CV-7 PTFe thread (GORE-TEX Suture, USA). The DMBA crystals (1 mg/25 g; Sigma-Aldrich, USA) were implanted in the distal pancreas. The implantation site was carefully checked to ensure there was no spillage of DMBA. The sutured pancreas and spleen were gently returned to the abdominal cavity. The incision was closed with 4–0 prolene suture lines. This procedure is illustrated in the (Additional file 1: Figure S1). The animals were subdivided into four groups: the control group (Con.), the aspirin group (ASP), the Lipitor group (LIP), and the combination of aspirin and Lipitor group (ASP + LIP). The mice were fed the appropriate drugs 2 weeks prior to surgery: water for the control group; 20 mg/kg aspirin daily for the aspirin group; 10 mg/kg Lipitor daily for the Lipitor group; and 20 mg/kg aspirin plus 10 mg/kg Lipitor daily for the combinational group. After the surgery, the same drug patterns were continued, and the drug dosages fed to the mice were equivalent to those used to prevent cardiovascular diseases and treat hyperlipemia clinically. Two months after surgery, the mice were sacrificed and the peripheral blood and pancreatic lesions were analyzed. Histological analysis for the pancreatic lesions was performed by two pathologists back-to-back.

### Carboxyfluorescein succinimidyl amino ester (CFSE) staining of Panc02 cells

CFSE was used to monitor proliferation of Panc02 cells in vitro. Breifly, the Panc02 cell suspension was diluted into 10^7^/ml and then the CFES/DMSO solution was added. The final working solution of CFSE is 2.5 μmol/l. 30 min (at the room temperature) later, DMEM medium with 10 % FCS was added to stop the reaction. And then wash three times with PBS. The stained Panc02 cells showed bright green under fluorescence microscope. The stained Panc02 cells were co-cultured with BMCs or RAW 264.7 in vitro for the further experiments.

### Chemotherapy in vivtro

For in vitro experiments of gemcitabine treatment, the Panc02 cells were incubated in high glucose DMEM with BMCs or RAW 274.7 supplemented with 10 % FCS and 1 % penicillin and streptomycin in the presence of 10 ng/ml of GM-CSF. The BMCs or RAW 264.7 were co-cultured with Panc02 at a 10 × 10^5^: 5 × 10^5^ ratio in 6-well-plates. Eighteen hours after incubation, the co-cultured cells were divided into 5 groups: the co-culture control group, the chemotherapy group (Chemo., gemcitabine, 20 μmol/l), the chemotherapy + aspirin group (Chemo. + ASP, gemcitabine, 20 μmol/l; aspirin 5 mmol/l), the chemotherapy + Lipitor group (Chemo. + LIP, gemcitabine, 20 μmol/l; Lipitor, 40 μmol/l), and the chemotherapy + aspirin + Lipitor group (Chemo. + ASP + LIP, gemcitabine, 20 μmol/l; aspirin 5 mmol/l; Lipitor, 40 μmol/l). The co-cultured cells were harvested 72 h after the drug treatment. Before harvesting, the prolifation of Panc02 cells were evaluated under fluoresence microscope. And then the co-cultured cells were prepared for FCM analysis (Accuri C6, BD, USA). The inflammatory cells in the co-culture were labeled as the CD45^+^ populations, and then the subpopulation in the CD45^+^ population were sorted and analyzed.

### Chemotherapy in vivo

To observe the effects of the combinational use of aspirin and Lipitor on the chemotherapy of pancreatic cancer in vivo, the Panc02 cell s.c implantation model was adopted. To establish the tumor bearing mouse model, 2 × 10^6^ Panc02 cells were subcutaneously implanted, which was previously determined to produce tumor masses that would reach 100–250 mm^3^ in 2 weeks after implantation. The mice were divided into five groups: the control group (Con.), the chemotherapy group (Chemo.), the chemotherapy + aspirin group (Chemo. + ASP), the chemotherapy + Lipitor group (Chemo. + LIP) and the chemotherapy + aspirin + Lipitor group (Chemo. + ASP + LIP). Two weeks prior to cell implantation, the mice were fed aspirin or Lipitor. Two weeks after cell implantation, the chemotherapy drug gemcitabine was administered 100 mg/kg intraperitoneal (i.p) injection once a week. The tumor volume was measured every 3 days using calipers. Twenty-four days after commencing chemotherapy, the tumor bearing mice were sacrificed and the peripheral blood and tumor tissue were analyzed.

### Statistics

All data were presented as the Mean ± SEM. The ANOVA, SNK-q test, paired student’s t-test and *Fisher* exact test were applied appropriately, and a 95 % confidence limit was considered to be significant, defined as *P* < 0.05. The IBM SPSS Statistics software 22.0 version and the Graphpad prism software 5.0 version were used for statistical analysis and drawing the graphs (Version 5.0, Graph-Pad Software, La Jolla, CA, USA).

## Results

### The dynamic changs of inflammatory cells in peripheral blood and pancreatic lesions in the process of pancreatic cancerogenesis

**Fig. 1:**
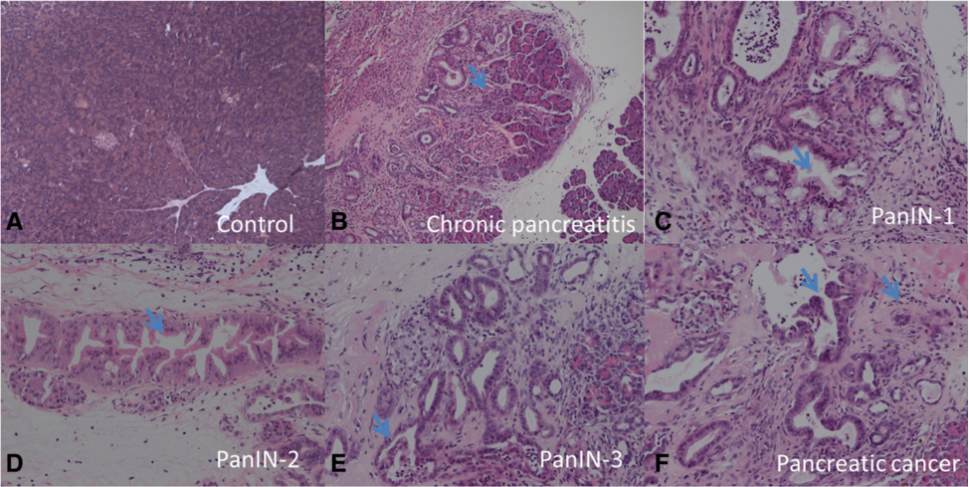
The representative pictures of DMBA-induced pancreatic cancerogenesis process from chronic pancreatitis (CP) to PanIN (pancreatic intradutal neoplasia) and invasive pancreatic cancer. **a** Normal pancreas, H&E, ×40; **b** chronic pancreatitis: the acinar atrophy and stroma hyperplasia, H&E, ×40. **c**. PanIN-1: the columnar cells with a papillary intraductal arrangements, H&E, ×100. **d** PanIN-2: the papillary pseudostratified and hyper chromatic lesions, H&E, ×100; **e** PanIN-3: the severe cellular atypia and cribriform architecture, H&E, ×100; **f** Invasive pancreatic cancer: the spare pancreatic cancer cells in the stroma, H&E, ×200. (Blue arrows indicate representative lesions)

Forty mice were used to establish the model of pancreatic cancerogenesis. Two months later, 8 mice died, among the survivors, 6 cases were chronic pancreatitis (CP), 5 cases were PanIN-1, 5 cases were PanIN-2, 7 cases were PanIN-3 and 9 cases were pancreatic cancer (PC) (Fig. 1). The total number of CD45^+^ inflammatory cells and the 11 subpopulations in peripheral blood and pancreatic lesions were analyzed by FCM. In peripheral blood, the total number of CD45^+^ inflammatory cells gradually increased during the process of pancreatic cancerogenesis. In peripheral blood, the total number of CD45^+^ inflammatory cells in PC was 12257.25 ± 4752.13/μl, compared to 8874.67 ± 3456.32/μl in PanIN-3, 6443.29 ± 2356.44/μl in PanIN-2, 5421.24 ± 2006.71/μl in PanIN-1, 5041.77 ± 2118.32/μl in CP and 4475.54 ± 1456.99/μl in control, respectively (PC: control, PC: CP, *P* < 0.01; PC: PanIN-1, PC: PanIN-2, *P* < 0.05; PanIN-3: control, PanIN-3:CP, PanIN-3: PanIN-1, *P* < 0.05; PanIN-2: control, *P* < 0.05). Then the propotions of subpopulations in CD45^+^ inflammatory cells were analyzed. The percentage of granulocyte in PC ((45.2 ± 5.76 %) was significantly higher than that of control ((30.25 ± 5.76 %), CP ((31.40 ± 5.77 %), PanIN-1 ((35.55 ± 6.78 %), PanIN-2 ((35.12 ± 7.66 %) (*P* < 0.05). As well, the percentage of MDSC in PC ((7.23 ± 1.53 %) was significantly higher than that of control ((1.25 ± 1.06 %), CP ((2.10 ± 1.57 %), PanIN-1 ((2.05 ± 1.78 %), PanIN-2 ((2.12 ± 1.06)%) and PanIN-3 ((5.12 ± 1.66)%) (PC: control, PC: CP, PC: PanIN-1, PC: PanIN-2, *P* < 0.01; PC: PanIN-3, *P* < 0.05; PanIN-3: PanIN-2, PanIN-3: PanIN-1; PanIN-3:CP, PanIN-3: control, *P* < 0.05), but the other inflammatory cell populations were not significantly different. Compared to the dynamic changes in peripheral blood, the dynamic changes in pancreatic lesions were more complicated, briely, the total number of CD45^+^ inflammatory cells and most of the subpopulations increased significantly with the development of pancreatic ccancerogensis. After analyzing the propotions of subpopulations in CD45^+^ inflammatory cells, the percentages of granulocyte, MDSC, macrophage, and M2 were significantly elevated with the progression of pancreatic cancerogenesis, in contrary, the percentages of helper T cell and cytotoxic T cell were significantly decreased. The detail was summarized in Table 1.

**Table 1:** The dynamic changes of the inflammatory cell populations in pancreatic lesions during pancreatic cancerogenesis (%, Mean ± SEM)

Cell population	Pancreatic lesions					
	Control (*n* = 6)	CP (*n* = 6)	PanIN-1 (*n* = 5)	PanIN-2 (*n* = 5)	PanIN-3 (*n* = 7)	PC (*n* = 9)
Inflammatory cells	15.50 ± 3.45	27.04 ± 4.75^*^	30.53 ± 5.57^*^	34.53 ± 5.45^*^	45.23 ± 5.40^**#&^	49.54 ± 6.91^**#&@^
Th						
	15.13 ± 3.34	15.02 ± 3.47	11.02 ± 3.46	10.44 ± 2.30	8.03 ± 2.40^*#^	8.67 ± 3.11^*#^
	2.15 ± 0.57	4.24 ± 0.94^*^	3.45 ± 1.01^*^	3.62 ± 0.54^*^	3.75 ± 2.40^*^	4.12 ± 0.93^*^
CTL	12.05 ± 3.55	12.51 ± 3.74	10.12 ± 2.13	7.11 ± 3.40	7.05 ± 2.05^*#^	6.04 ± 2.55^*#^
	1.74 ± 0.25	3.21 ± 0.78^*^	3.23 ± 1.01^*^	2.87 ± 0.48^*^	3.3 ± 1.00^*^	3.04 ± 0.68^*^
B cell						
	15.07 ± 3.52	16.40 ± 4.22	15.28 ± 3.00	16.24 ± 3.02	14.00 ± 3.25	11.34 ± 3.26
	2.12 ± 0.26	4.37 ± 1.01^*^	4.55 ± 0.87^*^	5.44 ± 0.75^*^	6.30 ± 1.24^*^	5.39 ± 1.27^*^
Granulocyte	35.56 ± 7.22	37.04 ± 5.78	40.52 ± 7.53	47.55 ± 5.07^*^	55.24 ± 7.30^*#^	60.24 ± 8.67^**##^
	5.27 ± 1.26	10.51 ± 2.33^*^	13.11 ± 2.46^*^	15.78 ± 3.61^**^	25.78 ± 3.89^**#&@^	31.12 ± 4.97^**##&@^
Mφ	12.22 ± 3.45	11.55 ± 3.22	14.28 ± 2.77	17.12 ± 4.54^#^	20.05 ± 4.43^*#^	22.15 ± 3.65^*#^
	1.58 ± 0.42	3.24 ± 0.60^*^	3.51 ± 0.89^*^	5.91 ± 1.10^**#^	9.18 ± 2.16^**##&&@^	12.11 ± 2.47^**##&&@^
DC						
	7.24 ± 2.25	8.46 ± 3.70	6.74 ± 4.22	10.86 ± 3.36	12.25 ± 4.57	11.23 ± 3.45
	0.97 ± 0.47	2.15 ± 0.45^*^	2.04 ± 0.47^*^	3.56 ± 1.01^*^	5.52 ± 1.46^**#&^	5.31 ± 1.89^**#&^
NK	6.10 ± 1.22	5.84 ± 2.33	5.42 ± 1.65	6.24 ± 2.94	5.54 ± 1.93	4.22 ± 1.58
	0.88 ± 0.24	1.68 ± 0.67^*^	1.98 ± 0.49^*^	2.27 ± 0.75^*^	2.51 ± 0.68^*^	2.29 ± 0.84^*^
NKT	3.01 ± 0.55	3.12 ± 0.97	2.01 ± 0.18	2.02 ± 0.65	2.76 ± 1.15	2.84 ± 1.44
	0.44 ± 0.21	0.91 ± 0.31	0.67 ± 0.12	0.75 ± 0.09	0.91 ± 0.21	0.83 ± 0.08
MDSC						
	5.24 ± 2.16	7.25 ± 2.55	9.25 ± 3.42^*^	14.14 ± 2.75^**#^	15.25 ± 3.55^**#^^&^	22.34 ± 4.22^**##&&@$^
	0.75 ± 0.15	1.79 ± 0.27^*^	2.75 ± 0.55^*^	3.64 ± 0.68^**#^	6.78 ± 1.45^**##&&@^	10.20 ± 2.07^**##&&@@$^
M1						
	8.54 ± 2.24	8.11 ± 2.78	7.55 ± 2.40	8.67 ± 3.70	9.44 ± 2.12	9.58 ± 2.55
	1.11 ± 0.27	2.41 ± 0.43^*^	2.55 ± 0.38^*^	2.97 ± 0.55^*^	4.27 ± 1.10^**#&^	4.68 ± 1.25^**#&@^
M2	2.72 ± 1.05	2.55 ± 1.17	5.01 ± 1.23^*^	5.15 ± 1.87^*^	6.94 ± 2.70^*#^	11.22 ± 2.77^**##&@$^
	0.29 ± 0.05	0.65 ± 0.10^*^	1.51 ± 0.42^**#^	2.25 ± 0.84^**#^	3.14 ± 0.42^**##&^	5.87 ± 1.28^**##&&@$^

The proportions of CD45^+^ inflammatory cells in all cells were compared. And then the proportions of different subpopulations in CD45^+^inflammatory cells (upper sub-row) and all cells (lower sub-row) were compared,respetively. *Th* helper T cells, *CTL* cytotoxic T lymphocyte, *Mφ* monocyte & macrophage, *DC* dendritic cells, *NK* natural killer cells, *NKT* natural killer T cells, *MDSC* myeloid derived suppressor cells, *M1* M1-polarized tumor associated macrophages, *M2* M2-polarized tumor assocaited macrophages. Compared with Control group, * *P* < 0.05, ** *P <* 0.01; Compared with CP (chronic pancreatitis), ^#^ *P* < 0.05, ^##^ *P* <0.01; Compared with PanIN-1 group, & *P* < 0.05,&& *P* < 0.01; Compared with PanIN-2, ^@^ *P* < 0.05, ^@@^ *P* < 0.01; Compared with PanIN-3, ^$^ *P* < 0.05, ^$$^ *P* < 0.01; ANOVA analysis, SNK-q test

### The dynamic changs of inflammatory cells in peripheral blood and tumor tissue after chemotherapy

After chemotehrapy, the total number of CD45^+^ inflammatory cells in peripheral blood was significantly decreased. The peripheral blood total number of CD45^+^ inflammatory cells in pancreatic cancer bearing mice without chemotherapy was 14358.76 ± 5656.22/μl, compared to 7449.7 4 ± 3237.46/μl in ones with chemotherapy. Further analysis of the subpopulations indicated that the percentages of dendritic cells (DC) and M2 were elevated and the percentage of MDSC was decreased in the peripheral blood. In tumor tissue, after chemotherapy, the propotion of CD45^+^ inflammatory cells in all cells seemed to be decresaed, however they were not statistically significant ((37.45 ± 6.98)%: (44.66 ± 7.32)%, *P* > 0.05). The percentages of Th, CTL and MDSC in CD45^+^ inflammatory cells were significantly decreased, in contrary,the percentage of B cell, macropahge, DC and M2 were significantly elevated. Then the propotions of the 11 subpopulations in total cells in tumor tissue were analyzed as well. The detail was summarized in Table 2.

**Table 2:** The changes of inflammatory cell populations in peripheral blood and tumor tissue after chemotherapy (*n* = 5, %, Mean ± SEM)

Population	Peripheral blood		Tumor tissue			
	Control	Chemotherapy	Control	Chemotherapy	Control	Chemotherapy
			in inflammatory cells		in all cells	
Th	12.21 ± 4.35	15.21 ± 3.37	15.26 ± 2.67	7.20 ± 2.00^*^	6.11 ± 1.07	2.25 ± 1.04^*^
CTL	9.45 ± 2.75	9.40 ± 2.75	10.53 ± 2.00	5.56 ± 1.57^*^	3.12 ± 0.73	1.01 ± 0.55^*^
B cell	25.54 ± 4.51	30.44 ± 5.23	10.70 ± 1.52	19.07 ± 2.56^*^	3.45 ± 1.01	6.28 ± 1.27^*^
Granulocyte	43.57 ± 6.72	37.56 ± 4.55	40.12 ± 6.41	38.56 ± 4.43	12.04 ± 3.27	10.44 ± 2.49
Mφ	10.11 ± 3.58	10.12 ± 2.46	16.61 ± 3.11	24.12 ± 4.12^*^	5.28 ± 0.76	8.14 ± 1.07^*^
DC	7.27 ± 2.12	16.45 ± 3.26^*^	8.13 ± 1.64	15.21 ± 2.12^*^	2.67 ± 1.22	4.77 ± 1.48^*^
NK	7.60 ± 1.12	6.55 ± 1.01	4.32 ± 1.12	3.31 ± 1.10	1.24 ± 0.52	1.05 ± 0.75
NKT	3.25 ± 0.44	2.27 ± 0.06	2.30 ± 0.24	3.32 ± 0.57	0.88 ± 0.16	0.75 ± 0.42
MDSC	25.22 ± 5.18	15.20 ± 3.10^*^	28.67 ± 3.32	18.75 ± 2.35^*^	7.44 ± 1.29	4.55 ± 0.92^*^
M1	9.67 ± 2.21	8.55 ± 2.01	7.36 ± 2.91	9.11 ± 2.12	2.46 ± 1.25	3.12 ± 1.09
M2	1.91 ± 1.58	5.11 ± 1.54^*^	10.97 ± 3.30	18.12 ± 3.56^*^	3.36 ± 0.70	5.98 ± 1.55^*^

*Th* helper T cells, *CTL* cytotoxic T lymphocyte, *Mφ* monocyte & macrophage, *DC* dendritic cells, *NK* natural killer cells, *NKT* natural killer T cells, *MDSC* myeloid derived suppressor cells, *M1* M1-polarized tumor associated macrophages *M2*, M2-polarized tumor assocaited macrophages. Paired Student t-test, ^*^ *P* < 0.05

### The roles of aspirin and Lipitor to regulate MDSC and TAM

**Fig. 2:**
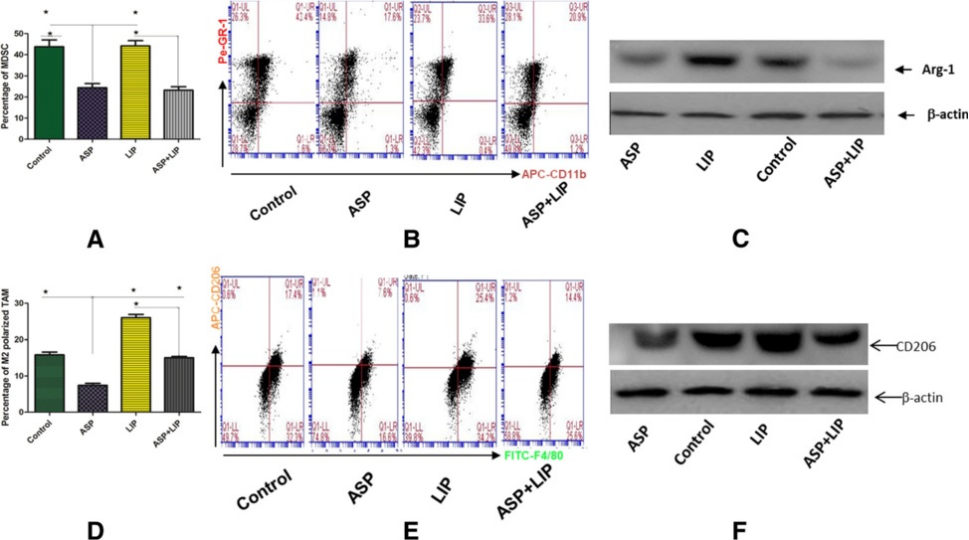
Aspirin inhibited pancreatic cancer-induced MDSC and M2 in vitro, however Lipitor promoted pancreatic cancer-induced M2. **a** The FCM results indicated that aspirin significantly inhibited pancreatic cancer-induced MDSC, however Lipitor did not obviously affect pancreatic cancer-induced MDSC. **b** The representative plots of FCM. **c** Western blotting of arginase-1 confirmed the results of FCM. **d** The FCM results indicated that aspirin significantly inhibited pancreatic cancer-induced M2, on the contrary, Lipitor obviously promoted pancreatic cancer-induced M2. **e** The representative plots of FCM. **f** Western blotting of CD206 onfirmed the results of FCM. Each experiment was performed in triplicats and the representative one was shown. ANOVA analysis, SNK-q test, ^*^ *P* < 0.05, ^**^ *P* < 0.01

The results from FCM indicated that aspirin significantly inhibited the expansion of MDSC and the M2, however Lipitor did not significantly affect MDSC, unexpected to us, it promoted the expansion of M2 signifiantly. Western blotting was used to detect, the specific effector of MDSC, arginas-1 (arg-1) and specific marker of M2 (CD206) as well. The results confirmed the results of FCM (Fig. 2).

### The effects of aspirin and Lipitor on the efficacy of gemcitabine in vitro

**Fig. 3:**
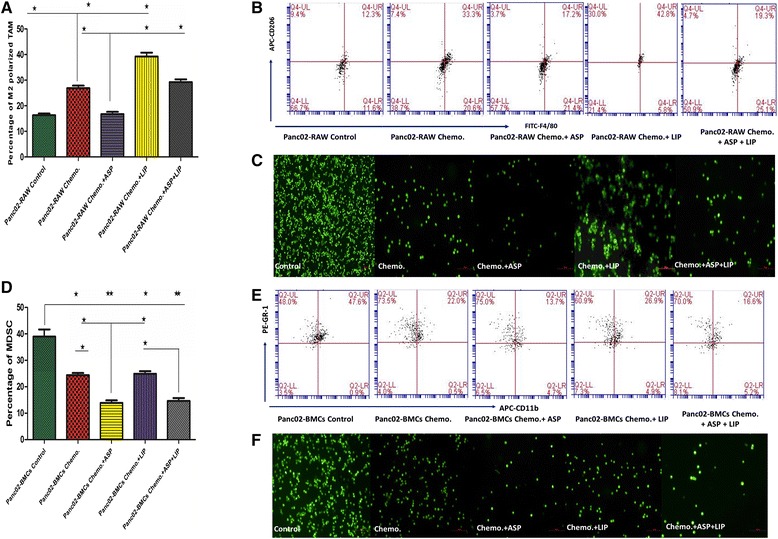
Aspirin augmented the efficacy of gemcitabine in vitro partially by inhibiting MDSC and M2, however Lipitor weakened the efficacy of aspirin and Lipitor by promoting M2. **a** The RAW264.7 macrophages and Panc02 cells were co-cultured, and gemcitabine was used for chemotherapy. The proportions of M2 (F4/80^+^ CD16/32^+^ CD206^+^) in CD45^+^ cells were detected by FCM. The results indicated that gemcitabine induced M2, aspirin inhibited M2, however Lipitor promoted gemcitabine-induced M2. **b** The representative plots of FCM. **c** The CFSE stained Panc02 cells were observed under fluorescent microscope (×100), the results indicated the Panc02 cells were sensitive to gemcitabine, aspirin augmented the efficacy of gemcitabine, however Lipitor weakened the efficacy of aspirin and gemcitabine. **d** The BMCs (bone marrow cells) and Panc02 cells were co-cultured, and gemcitabine was used for chemotherapy as well. The proportions of MDSC (CD11b^+^ Gr-1^+^) in CD45^+^ cells were detected by FCM. The results indicated that gemcitabine inhibited MDSC, aspirin inhibited MDSC as well, but Lipitor did not significantly affect MDSC. **e** The representative plots of FCM. **f** The CFSE stained Panc02 cells were observed under fluorescent microscope (×100), the results indicated aspirin augmented the efficacy of gemcitabine, however Lipitor did not significantly affect the efficacy of aspirin and gemcitabine. Each experiment was performed in triplicats and the representative one was shown. ANOVA analysis, SNK-q test, ^*^ *P* < 0.05, ^**^ *P* < 0.01

The CFSE-labeled Panc02 cancer cells were cultured with BMCs or RAW264.7 cells and then gemcitabine was used for chemotherapy. FCM was used to detect the MDSC and M2. Fluorescence microscopy was used to indicate the density of CFSE-labeled Panc02 cells. After chemotherapy, the percentage of M2 significantly increased, and aspirin inhibited the expansion of MDSC and M2, but Lipitor did not significantly inhibit MDSC, instead it promoted M2. The density of pancreac cancer cells indicated that Panc02 cells were sensitive to gemcitabine, and aspirin enhanced the efficacy of gemcitabine, however Lipitor significantly weakened the efficacy of gemcitabine and aspirin by promoting M2 (Fig. 3).

### The effects of aspirin and Lipitor on the efficacy of gemcitabine in vivo

**Fig. 4:**
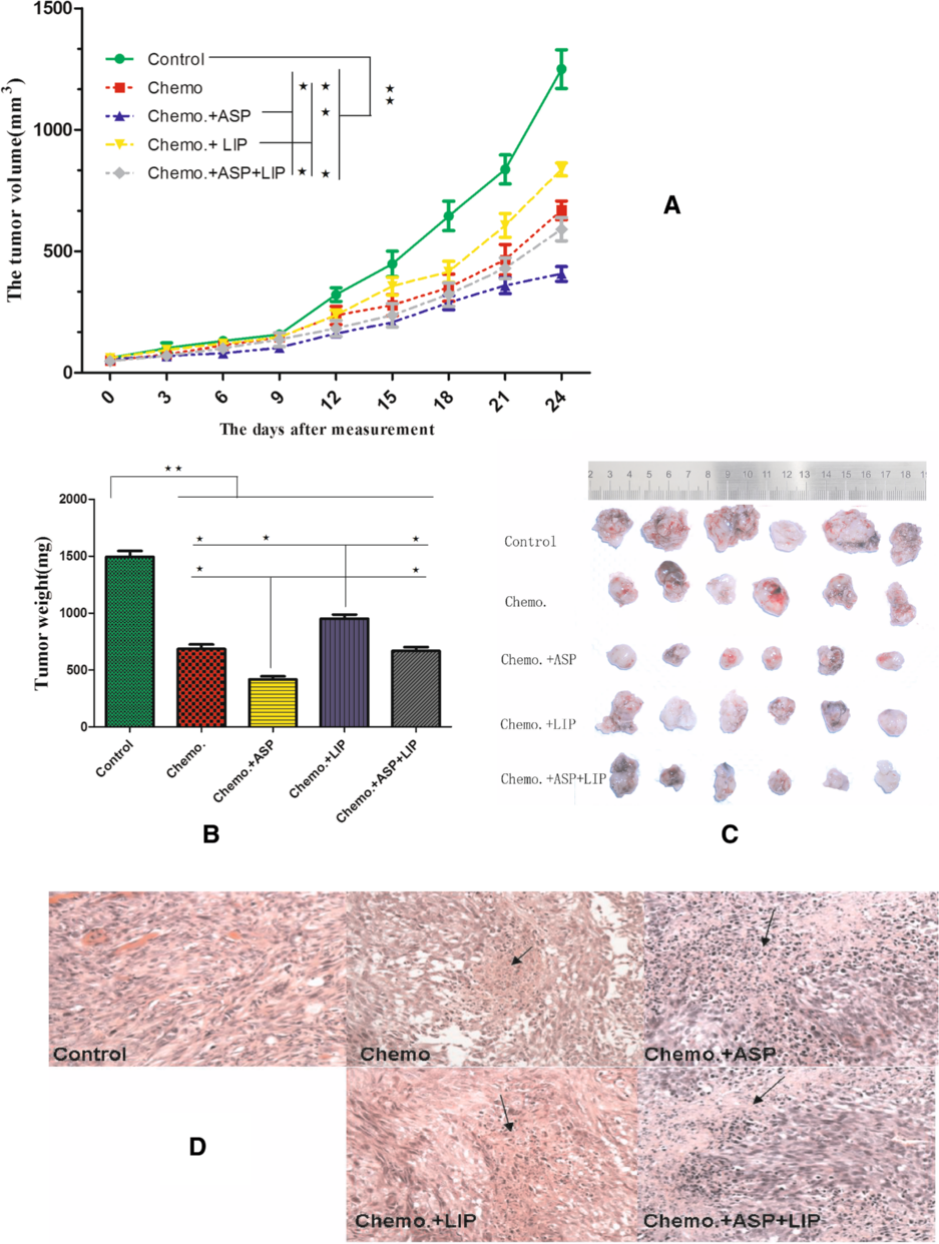
Aspirin augmented the efficacy of gemcitabine in vivo, however Lipitor weakened the efficacy of gemcitabine. **a** Panc02 pancreatic cancer cells were subcutanously implanted in the immunocompetent mice and then gemcitabine was intrperitoneally injected for chemotherapy. (**a**) The tumor growth curve indicated that aspirin augmented the efficacy of gemcitabine, on the contrary, Lipitor weakened the efficacy of aspirin and gemcitabine. **b** The final tumor weight confired the results of the tumor growth curver. **c** The general figure of fresh tumors were shown. **d** H&E staining of the necrosis lesions also confirmed the efficacy of aspirin and gemcitabine (×200). Each experiment was performed in triplicats and the representative one was shown. . ANOVA analysis, SNK-q test, ^*^ *P* < 0.05, ^**^ *P* < 0.01

**Fig. 5:**
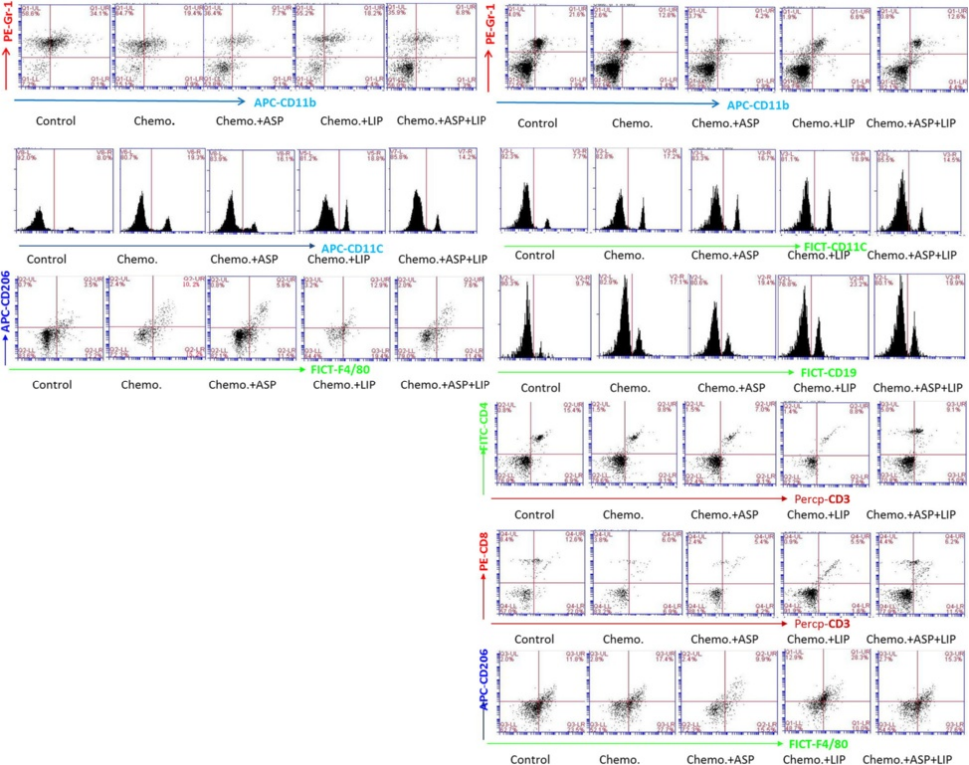
The representative plots of the dynamic changes of inflammatory cells in peripheral blood and tumor tissue after chemotherapy. Left plots from upper to lower were MDSC, DC and M2 in peripheral blood respectively. Right figures from upper to lower were MDSC, DC, B cells, Th, CTL and M2 in tumor tissue respectively. Each experiment was performed in triplicats and the representative one was shown

**Fig. 6:**
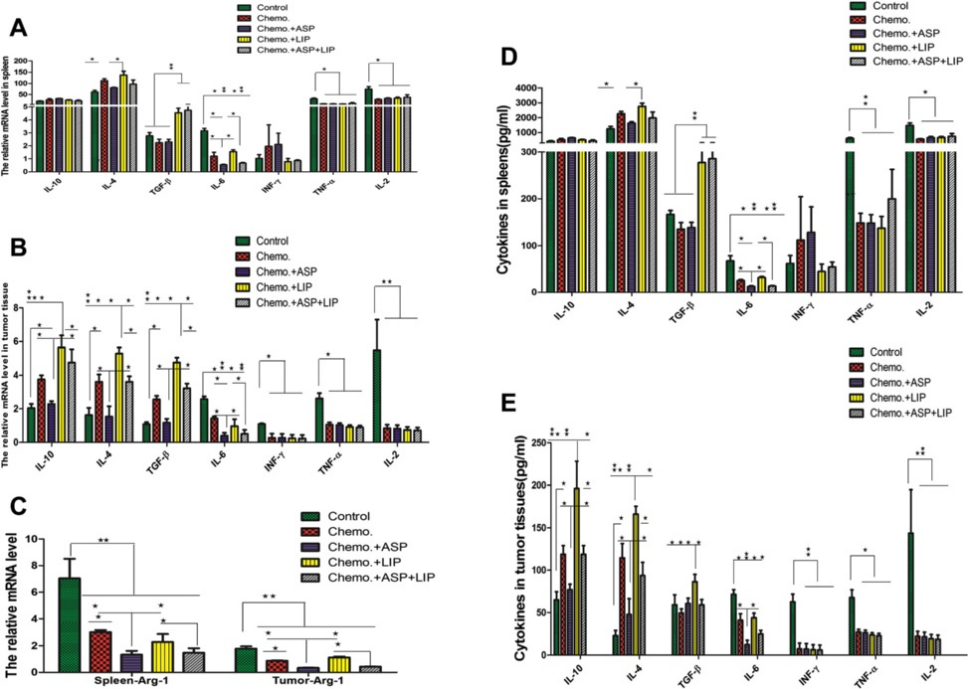
Gemcitabine induced systemic and intratumoral Th2 biased cytokine environment, aspirin weakened these effects of gemcitabine, however Lipitor augmented these effects. **a**. The cytokines in spleen were measured by real time RT-PCR. The results indicated that gemcitabine reduced the levels of IL-6, TNF-α and IL-2, however it increaed the levels of IL-4. Lipitor increased the levels of IL-4 and TGF-β. Aspirin lowered down IL-6. And the results were confirmed by the elisa tests (**d**). **b**. The cytokines in tumor tissues were measured by real time RT-PCR. The results indicated that gemcitabine reduced the intratumoral levels of IL-6, INF-γ, TNF-α and IL-2, however it elevated the levels of IL-4, IL-10 and TGF-β. Lipitor increased the levels of IL-4, IL-10 and TGF-β. Aspirin lowered down IL-4, IL-10, TGF-β and IL-6. And the results were confirmed by the elisa tests (**e**). **c**. The arginase in spleens and tumor tissues were detected by real time RT-PCR. The results confirmed the results of FCM, that is, gemcitabine inhibited MDSC, aspirin inhibited MDSC as well, however Lipitor did not affect MDSC significantly. Each experiment was performed in triplicats and the representative one was shown. . ANOVA analysis, SNK-q test, ^*^ *P* < 0.05, ^**^ *P* < 0.01

Subtaneous implantation of Panc02 cells wer used to test the efficacy of gemcitabine and the roles of aspirin and Lipitor in vivo. The tumor growth curve indicated that the similar results like the in vitro results, that is, aspirin had synergic roles with gemcitabine, however Lipitor attenuated the efficacy of gemcitabine and aspirin (Fig. 4). The inflammatory cell populations in tumor tissue and peripheral blood of the different groups were analyzed. In peripheral blood, aspirin had synergic roles to inhibit MDSC with gemcitabine and as well it inhibited gemcitabine- promoted M2, however Lipitor significantly enhanced the roles of gemcitabine to promote M2 (Table 3, Fig. 5). In tumor tissue, gemcitabine induced accumulation of B cells,DC and M2, on the contrary, it reduced the infiltrations of Th, CTL and MDSC. Aspirin had synergic roles with gemcitabine to reduce MDSC, and it also weakened gemcitabine-induced M2, however Lipitor augmented gemcitabine-induced M2 (Table 4, Fig. 5). Later we anaylzed the cytokines in spleen and tumor tissue by real time RT-PCR and elisa tests. We also confirmed that the arginase-1 in spleen and tumor tissue were reduced by gemcitabine and aspirin. Gemcitabine induced significant systematic and local intratumoral Th2 cytokine environment which could be the curcial factors to promote M2, aspirin inhibited theses effects of gemcitabien, however Lipitor augmetned these effects of gemcitabine (Fig. 6).

**Table 3:** The changes of the inflammatory cell populations in peripheral blood after chemotherapy (*n* = 6, %, mean ± SEM)

Cell population	Peripheral blood				
	Control	Chemo.	Chemo. + ASP	Chemo. + Lip	Chemo. + ASP + LIP
Th	13.46 ± 3.33	12.01 ± 3.48	13.12 ± 2.16	12.98 ± 2.01	12.01 ± 1.30
CTL	10.44 ± 2.65	9.22 ± 2.65	8.11 ± 2.05	10.11 ± 3.44	9.40 ± 1.75
B cells	27.52 ± 5.01	26.04 ± 5.12	31.01 ± 5.01	22.24 ± 4.01	24.88 ± 6.27
Granulocyte	45.55 ± 7.71	32.33 ± 5.75	32.55 ± 4.66	35.46 ± 4.44	38.22 ± 4.77
Mφ	13.12 ± 3.44	17.11 ± 2.52	9.11 ± 2.05	13.11 ± 4.08	14.01 ± 3.58
DC	7.34 ± 2.06	15.42 ± 2.34^*^	17.77 ± 4.37^*^	15.88 ± 1.34^*^	18.24 ± 2.07^*^
NK	6.56 ± 2.18	7.50 ± 2.04	5.44 ± 1.25	6.80 ± 1.22	7.70 ± 1.22
NKT	2.29 ± 1.40	3.01 ± 1.26	3.01 ± 0.67	3.20 ± 0.67	3.80 ± 1.04
MDSC	23.21 ± 4.29	14.70 ± 3.14^*^	7.20 ± 2.45^**^^#^	16.01 ± 3.24^*&^	9.28 ± 1.24^**^^#^
M1	7.87 ± 2.88	10.85 ± 1.45	8.58 ± 3.44	9.05 ± 2.75	11.57 ± 2.76
M2	2.91 ± 1.58	7.11 ± 1.54*	3.01 ± 1.05^#^	11.01 ± 2.04** ^#^	5.22 ± 1.77

*Th* helper T cells, *CTL* cytotoxic T lymphocyte, *Mφ* monocyte & macrophage, *DC* dendritic cells, *NK* natural killer cells, *NKT* natural killer T cells, *MDSC* myeloid derived suppressor cells, *M1* M1-polarized tumor associated macrophages, *M2* M2-polarized tumor assocaited macrophages. Compared with Control group, * *P* < 0.05, ** *P* < 0.01; Compared with Chemo.group, ^#^ *P* < 0.05, ^##^ *P* < 0.01; Compared with Chemo. + ASP group, & *P* < 0.05,&& *P* < 0.01; ANOVA analysis, SNK-q test

**Table 4:** The changes of the inflammatory cell populations in tumor tissue after chemotherapy (*n* = 6, %, Mean ± SEM)

Population	Tumor tissue				
	Control	Chemo.	Chemo. + ASP	Chemo. + Lip	Chemo. + ASP + LIP
Inflammatory cells	45.57 ± 6.55	41.01 ± 6.00	38.50 ± 7.43	35.50 ± 4.77	36.55 ± 6.79
Th	14.24 ± 2.54	8.10 ± 2.55^*^	8.24 ± 2.22^*^	7.12 ± 2.44^*^	7.44 ± 2.45^*^
	6.71 ± 1.33	3.54 ± 1.55^*^	3.27 ± 1.25^*^	2.75 ± 0.87^*^	2.72 ± 1.01^*^
CTL	9.52 ± 1.05	5.57 ± 1.24^*^	6.22 ± 1.57^*^	6.11 ± 1.47^*^	6.22 ± 2.45^*^
	4.05 ± 0.74	1.95 ± 0.57^*^	2.32 ± 1.05^*^	1.87 ± 0.48^*^	2.01 ± 1.14^*^
B cell	10.11 ± 2.54	18.27 ± 3.54^*^	18.71 ± 1.33^*^	17.70 ± 2.02^*^	19.07 ± 2.44^*^
	4.01 ± 1.11	7.32 ± 1.76^*^	6.91 ± 1.05^*^	6.45 ± 1.02^*^	7.01 ± 1.27^*^
Granulocyte	31.13 ± 5.65	26.54 ± 4.32	27.58 ± 4.76	27.79 ± 3.33	25.50 ± 4.50
	12.70 ± 2.95	10.11 ± 2.75	9.97 ± 2.64	9.45 ± 1.45	9.57 ± 2.27
Macrophage	17.62 ± 3.26	25.17 ± 3.22^*^	17.03 ± 2.55^#^	32.04 ± 4.77^**#&^	25.88 ± 3.46^*&^
	7.45 ± 1.81	10.25 ± 2.02	6.52 ± 1.57^#^	13.14 ± 1.68^*&^	9.69 ± 1.78^&^
DC	7.17 ± 1.33	14.24 ± 2.32^*^	17.77 ± 2.34^*^	16.24 ± 1.66^*^	15.01 ± 1.68^*^
	3.02 ± 0.56	5.87 ± 1.02^*^	6.75 ± 1.42^*^	5.64 ± 0.56^*^	5.42 ± 0.78^*^
NK	5.34 ± 1.64	4.32 ± 2.17	4.72 ± 1.02	4.44 ± 1.13	4.41 ± 1.02
	1.34 ± 0.43	1.27 ± 0.33	1.12 ± 0.24	1.04 ± 0.35	0.97 ± 0.28
NKT	2.34 ± 1.26	3.35 ± 1.58	2.55 ± 0.88	2.24 ± 0.15	4.02 ± 1.21
	0.82 ± 0.45	1.05 ± 0.37	0.96 ± 0.46	0.81 ± 0.17	1.11 ± 0.48
MDSC	19.76 ± 4.31	11.65 ± 3.25^*^	6.34 ± 1.11^**#^	11.57 ± 1.89^*^^&^	7.50 ± 1.45^**#@^
	9.05 ± 2.37	4.75 ± 1.64^*^	2.88 ± 0.74^**#^	4.28 ± 0.58^*&^	2.70 ± 0.84^**#@^
M1	9.34 ± 2.76	10.22 ± 2.66	10.23 ± 2.67	10.23 ± 3.12	9.37 ± 2.45
	3.72 ± 1.43	4.11 ± 1.23	3.56 ± 1.26	3.45 ± 1.24	3.03 ± 1.05
M2	11.98 ± 3.26	18.22 ± 3.37^*^	15.60 ± 4.34^*#^	27.89 ± 4.58^**#&&^	22.11 ± 2.76^*&^
	4.46 ± 1.57	7.68 ± 1.85^*^	5.81 ± 2.08	10.79 ± 1.75^**#&^	7.78 ± 1.64^*^

The proportions of CD45^+^ inflammatory cells in all cells were compared. And then the proportions of different subpopulations in CD45^+^inflammatory cells (upper sub-row) and in all cells (lower sub-row) were compared. *Th* helper T cells, *CTL* cytotoxic T lymphocyte, *DC* dendritic cells, *NK* natural killer cells, *NKT* natural killer T cells, *MDSC* myeloid derived suppressor cells, *M1* M1-polarized tumor associated macrophages, *M2* M2-polarized tumor assocaited macrophages. Compared with Control group, * *P* < 0.05, ** *P <* 0.01; Compared with Chemo.group, ^#^ *P* < 0.05, ^##^ *P* <0.01; Compared with Chemo. + ASP group, & *P* < 0.05,&& *P* < 0.01; Compared with Chemo. + LIP, ^@^ *P* < 0.05, ^@@^ *P* < 0.01; ANOVA analysis, SNK-q test

### The efffects of aspirin and Lipitor on preventing pancreatic cancerogensis

One hundred sixty mice were used to establish the model of pancreatic cancerogenesis. Two months later, the mice were sacrified. Among the four grups, the incidence of pancreatic cancer in the ASP group was the lowest (12.5 %) compared to the other groups (*P* < 0.05). When Lipitor was used with aspirin, the protective roles of aspirin was weakened (ASP: ASP + LIP, 12.5 %: 25 %, *P* < 0.05). The detail was summarized in Table 5.

**Table 5:** The distribution of the pancreatic lesions induced by DMBA

					PanIN		
	n	Death	CP	I	II	III	CA
Control	40	5	3	6	7	8	11
ASP	40	3	11	10	8	3	5^*^
LIP	40	5	3	4	7	7	14
ASP + LIP	40	4	5	8	5	8	10

Fisher extact test,*P < 0.05

## Discussion

Chemopreventive strategies for pancreatic cancerogenesis and to improve the efficacy of gemcitabine are of great value. Pancreatic cancer is a well known inflammatory malignancy with exclusive intratumoral fibrosis and abundant infiltration of immune cells. Increasing evidence suggests that inflammations influence tumor development and the interactions of inflammatory cells with chemotherapies are more complicated than that ever been imagined [15–19]. To address these questions, we tracked the dynamic changes of pan-inflammatory cell populations during the course of pancreatic cancerogenesis in a cancerogen DMBA-induced mouse model of pancreatic cancerogensis and a subtutanous tumor implantation model. This DMBA-induced pancreatic cancerogenesis model induces the same characteristic stages of neoplasia in the evolution of ductal pancreatic cancer as observed in humans, and the *K-ras* mutations occur progressively in the ladder of cancerogenesis, similar to human pancreatic cancer [20, 21]. Compared with the genetically engineered mouse model, this model saves time, animals and cost and can mimic the whole pancreatic cancerogenesis process from PanIN to invasive cancer in a shorter time period. We found that disease progression from normal pancreatic tissue, chronic pancreatitits, PanIN to pancreatic cancer was accompanied by a progressive infiltration of CD45^+^ inflammatory cells, in which the percentages of granulocyte and macrophages were in prevalence comprising nearly half of the inflammatory cells at the inception of pancreatic cancerogenesis and dramatically increased, on the contrary, the proportions of Th and CTL significantly decreased. The accumulated granulocytes gradually turn into an immature immunosuppressive phenotye MDSC, and the macrophages polarized into a tumor-supporting phenotype M2. The accumulated MDSC and M2 with reduction of Th cells and CTL indicated an immunosuppressive microenvironment at the beginning of the pancreatic cancerogenesis. The elevated MDSC in peripheral blood of patients with pancreatic cancer was reported to be positively related with tumor stage and negatively related with prognosis [22, 23]. In a gene engineered pancreatic cancerogenesis murine mode, the MDSC was found to accumulate at the inception of cancerogensis [24]. The microenvironment of pancreatic cancer can activate the STAT3 (signal transducers and activators of transcription 3) signal pathway in MDSC, and then the activated MDSC can maintain the pancreatic cancer stem cells [25, 26], and this feedback potentially could promote pancreatic cancerogenesis and affect the efficacy of chemotherapy. Macrophages in tumor can be induced to be an alternatively activated M2 phenotype mainly by the Th2 cytokine environment, which has potential immunosuppressive roles and some other tumor supporting roles [19]. Higher intratumoral infiltration of M2 predicted poor prognosis of pancreatic cancer [27, 28]. M2 can promote epithelial-mesenchymal transition in pancreatic cancer cells, partially through TLR4/IL-10 signaling pathway [29]. This murine Panc02 pancreatic cancer was highly sensative to gemcitabine. After chemotherapy, gemcitabine obviously induced a Th2 biased cytokine microenvironment characterized by higher level of interleukin-4 (IL-4), interleukin-10 (IL-10) and TGF-β, as well the percentages of B cells, dendritic cells (DC) and M2 in peripheral blood and tumor tissue were significantly elevated, on the contrary, the percentages of intratumoral Th cells and CTL, and that of MDSC in peripheral blood and tumor tissue were decreased as well. Besides tumor cell necrosis, gemcitabine also could induce immunogenic death of pancreatic cancer cells [30], the gemcitabine-induced release of immunogenic particles of pancreatic cancer cells could be the trigger for the accumulation of dendritic cells. The lysate pancreatic cancer stem cells and vaccine-senetised dendritic cells have obvious synergic roles with gemcitabine [31]. Gemcitabine can directy inhibit the expansion of MDSC in murine breast cancer models [32] and in this study, we also found gemcitabine inhibited expansion of MDSC in bothe tumor tissue and peripheral blood. We also found after chemotherapy, there were more intratumoral B cells. The B cells impaired the efficacy of platinum and taxol to squamous cancer by altering chemokine experssion of macrophages that foster infiltration of activated CD8^+^ T cells via CCR5-dependent mechanisms [33]. Th2 cytokine-induced IgG4 positive B cells can dampen the activity of macrophages by competitively binding to Fc receptor [34], however when activated by CD40 signal pathway, macrophages can significantly improve the efficacy of gemcitabine [35]. Gemcitabine dramatically induced M2 expansion in this study. M2 can mediate gemcitabine resistance of pancreatic adenocarcinoma by upregulating cytidine deaminase. Higher level of M2 predicted a poor prognosis of pancreatic cancer after chemotherapy [36, 37]. So the accumulated intratumoral B cells induced by gemcitabine could possibly contribute to secendary drug resistance. After thoroughly scanning of the dynamic changes of inflammatory cells, we found MDSC and M2 were potential targets of great values for pancreatic cancer prevention and imrpoving chemotherapeutic efficacy of gemcitabine.

Aspirin and atorvastatin are always prescribed together to treat or prevent cardio-cerebral vascular diseases maily by regulation of inflammations, and they have been reported to have plausible anti-tumor roles. We initially attempted to develop novel strategies for pancreatic cancer prevention and for improving the efficacy of gemcitabine by means of combining of these two drugs partially by regulation of cancer related inflammations. The COX/PGE axis has been reported to play important roles to induce MDSC and M2, as an effective inhibitor of this axis, aspirn has been reported to inhibit the expansion of MDSC [8], and in this study we also found aspirin substantially inhibited the expansion of MDSC and M2 polarization in pancreatic cancer. Lipitor has been reporetd to inhibit the activation of macrophages in several ways. Lipitor suppresses inflammatory response induced by oxLDL through inhibition of ERK phosphorylation expression in murine macrophages [10]. Lipitor could induce LPS-mediated MMP experssion by inhibiting geranylgeranyl pyrophosphate and regulating phosphorylation of ERK and CREB, and MMP-9 is a marker for M2 [38]. It can also attenuates TNF-α production via Heme Oxygenase-1 Pathway in LPS-stimulated RAW264.7 macrophages,and TNF-α is a marker of M1 [39]. In this study, Lipitor significantly promoted the expansion of M2 in pancreatic cancer. Recently, a study reporetd that Lipitor promoted human monocyte differentiation toward alternative M2 macrophages via p38 MAPK-dependent PPARγ activation in vitro,envn if, in that study, the M2 was not induced by pancreatic cancer, it could be also a strong support to our findings in this paper [40]. When tested for their roles to prevent cancerogenesis and to improve the efficacy of gemcitabine, aspirin had substantial positive roles to prevent cancerogenesis and improve chemotherapeutic efficacy partially by inhibiting MDSC and M2, however Lipitor weakened the efficacy of aspirin and gemcitabine partially by promoting M2. Three large population-based studies did not support that Lipitor, administered at doses used to manage hypercholesterolaemia, could reduce the risk of pancreatic cancer as well [12, 41, 42]. Some studiese have even proposed that Lipitor are potential cancerogens. In a study conducted on 88,125 cases and 362,254 controls, long-term intake of Lipitor was associated with a significantly increased risk of development and recurrence of bladder and lung cancer [43, 44]. Pastore et al. [45] found that long-term treatment with aspirin in patients with non-muscle-invasive bladder cancer might play a role on reducing the risk of tumor recurrence. In contrast, statins and combination treatment groups showed increased recurrence rates. Several statins, have been found to be carcinogenic in rodents in doses that produce blood concentrations of the drugs similar to those attained in treating patients [46]. Our results also did not show any potential benefit of Lipitor to prevent pancreatic cancer and to improve chemotherapeutic efficacy of gemcitabine, unexpected to us, it even attenuated the positive roles of aspirin and gemcitabine. We suggest that aspirin alone could be more effective to prevent cancerogenesis and improve efficacy of gemcitabine.

## Conclusions

In conclusion, our present study demonstrates that 1) MDSC and M2 dramatically accumulate in the process of pancreatic cancerogenesis, first the time, we found gemcitabine could induce M2 which could be potential mechanism for secendary drug-resistance; 2) Aspirin could inhibit the expansion of MDSC and M2, but Lipitor promote M2; 3) Aspirin has substantial roles to prevent pancreatic cancerogenesis and improve the efficacy of gemcitabine partially by inhibiting MDSC and M2, when used in combination, Lipitor attenuates the postive roles of aspirin and gemcitabine partially by promoting M2.

## Additional file

Additional file 1: Figure S1.
**The illustration of the procedures of DMBA-induced pancreatic cancerogenesis model.** **Table S1.** The list of antibodies. **Table S2.** The list of the primers of the real time RT-PCR. (DOCX 2437 kb)

## Abbreviations

Arg- 1: arginase 1
ASP: aspirin
BMCs: bone marrow cells
CSFE: carboxyfluorescein succinimidyl amino ester
COX: cyclooxygenase
DMBA: dimethylbenzanthracene
DMEM: dulbecco’s modified eagle medium
DMSO: dimethyl sulfoxide
FCM: flow cytometry
FCS: fetal calf serum
Gem: gemcitabine
GM-CSF: granulocyte macrophage colony stimulating factor
GAPDH: glyceraldehyde 3-phosphate dehydrogenase
IL-2/4/6/10: interleukin-2/4/6/10
INF-γ: interferon-γ
i.p: intraperitoneal
LIP: Lipitor
MDSC: myeloid derived suppressor cells
PBS: phosphate buffered saline
s.c: subcutaneous
PEG: prostaglandin
STAT3: signal transducers and activators of transcription 3
TGF-β: transforming growth factor-β
TNF-α: tumor necrosis factor-α
TAM: tumor associated macrophages

## References

[1] DeSantis CE, Lin CC, Mariotto AB, Siegel RL, Stein KD, Kramer JL (2014). Cancer treatment and survivorship statistics, 2014. CA Cancer J Clin.

[2] Hruban RH, Adsay NV, Albores-Saavedra J, Compton C, Garrett ES, Goodman SN (2001). Pancreatic intraepithelial neoplasia: a new nomenclature and classification system for pancreatic duct lesions. Am J Surg Pathol.

[3] Sahoo RK, Kumar L (2014). Albumin-bound paclitaxel plus gemcitabine in pancreatic cancer. N Engl J Med.

[4] Erkan M, Hausmann S, Michalski CW, Fingerle AA, Dobritz M, Kleeff J (2012). The role of stroma in pancreatic cancer: diagnostic and therapeutic implications. Nat Rev Gastroenterol Hepatol.

[5] Karakhanova S, Link J, Heinrich M, Shevchenko I, Yang Y, Hassenpflug M (2015). Characterization of myeloid leukocytes and soluble mediators in pancreatic cancer: importance of myeloid-derived suppressor cells. Oncoimmunology.

[6] Gardian K, Janczewska S, Olszewski WL, Durlik M (2012). Analysis of pancreatic cancer microenvironment: role of macrophage infiltrates and growth factors expression. J Cancer.

[7] Kobayashi M, Shimodaira S, Nagai K, Ogasawara M, Takahashi H, Abe H (2014). Prognostic factors related to add-on dendritic cell vaccines on patients with inoperable pancreatic cancer receiving chemotherapy: a multicenter analysis. Cancer Immunol Immunother.

[8] Carlson LM, Rasmuson A, Idborg H, Segerstrom L, Jakobsson PJ, Sveinbjornsson B (2013). Low-dose aspirin delays an inflammatory tumor progression in vivo in a transgenic mouse model of neuroblastoma. Carcinogenesis.

[9] Lee DK, Park EJ, Kim EK, Jin J, Kim JS, Shin IJ (2012). Atorvastatin and simvastatin, but not pravastatin, up-regulate LPS-induced MMP-9 expression in macrophages by regulating phosphorylation of ERK and CREB. Cell Physiol Biochem.

[10] Shao Q, Shen LH, Hu LH, Pu J, Jing Q, He B (2012). Atorvastatin suppresses inflammatory response induced by oxLDL through inhibition of ERK phosphorylation, IkappaBalpha degradation, and COX-2 expression in murine macrophages. J Cell Biochem.

[11] Wender RC (2015). Aspirin and NSAID chemoprevention, gene-environment interactions, and risk of colorectal cancer. JAMA.

[12] Bonovas S, Filioussi K, Sitaras NM (2008). Statins are not associated with a reduced risk of pancreatic cancer at the population level, when taken at low doses for managing hypercholesterolemia: evidence from a meta-analysis of 12 studies. Am J Gastroenterol.

[13] Nielsen SF, Nordestgaard BG, Bojesen SE (2012). Statin use and reduced cancer-related mortality. N Engl J Med.

[14] Liu Q, Zhang M, Jiang X, Zhang Z, Dai L, Min S (2011). miR-223 suppresses differentiation of tumor-induced CD11b(+) Gr1(+) myeloid-derived suppressor cells from bone marrow cells. Int J Cancer.

[15] Keklikoglou I, De Palma M (2014). Cancer: Metastasis risk after anti-macrophage therapy. Nature.

[16] Wynn TA, Chawla A, Pollard JW (2013). Macrophage biology in development, homeostasis and disease. Nature.

[17] Lavin Y, Winter D, Blecher-Gonen R, David E, Keren-Shaul H, Merad M (2014). Tissue-resident macrophage enhancer landscapes are shaped by the local microenvironment. Cell.

[18] Qian BZ, Pollard JW (2010). Macrophage diversity enhances tumor progression and metastasis. Cell.

[19] De Palma M, Lewis CE (2013). Macrophage regulation of tumor responses to anticancer therapies. Cancer Cell.

[20] Kimura K, Satoh K, Kanno A, Hamada S, Hirota M, Endoh M (2007). Activation of Notch signaling in tumorigenesis of experimental pancreatic cancer induced by dimethylbenzanthracene in mice. Cancer Sci.

[21] Guo JC, Li J, Yang YC, Zhou L, Zhang TP, Zhao YP (2013). Oligonucleotide microarray identifies genes differentially expressed during tumorigenesis of DMBA-induced pancreatic cancer in rats. PLoS One.

[22] Markowitz J, Brooks TR, Duggan MC, Paul BK, Pan X, Wei L (2015). Patients with pancreatic adenocarcinoma exhibit elevated levels of myeloid-derived suppressor cells upon progression of disease. Cancer Immunol Immunother.

[23] Khaled YS, Ammori BJ, Elkord E (2014). Increased levels of granulocytic myeloid-derived suppressor cells in peripheral blood and tumour tissue of pancreatic cancer patients. J Immunol Res.

[24] Zhao F, Obermann S, von Wasielewski R, Haile L, Manns MP, Korangy F (2009). Increase in frequency of myeloid-derived suppressor cells in mice with spontaneous pancreatic carcinoma. Immunology.

[25] Panni RZ, Sanford DE, Belt BA, Mitchem JB, Worley LA, Goetz BD (2014). Tumor-induced STAT3 activation in monocytic myeloid-derived suppressor cells enhances stemness and mesenchymal properties in human pancreatic cancer. Cancer Immunol Immunother.

[26] Cui TX, Kryczek I, Zhao L, Zhao E, Kuick R, Roh MH (2013). Myeloid-derived suppressor cells enhance stemness of cancer cells by inducing microRNA101 and suppressing the corepressor CtBP2. Immunity.

[27] Kurahara H, Shinchi H, Mataki Y, Maemura K, Noma H, Kubo F (2011). Significance of M2-polarized tumor-associated macrophage in pancreatic cancer. J Surg Res.

[28] Ino Y, Yamazaki-Itoh R, Shimada K, Iwasaki M, Kosuge T, Kanai Y (2013). Immune cell infiltration as an indicator of the immune microenvironment of pancreatic cancer. Br J Cancer.

[29] Liu CY, Xu JY, Shi XY, Huang W, Ruan TY, Xie P (2013). M2-polarized tumor-associated macrophages promoted epithelial-mesenchymal transition in pancreatic cancer cells, partially through TLR4/IL-10 signaling pathway. Lab Invest.

[30] Angelova AL, Grekova SP, Heller A, Kuhlmann O, Soyka E, Giese T (2014). Complementary induction of immunogenic cell death by oncolytic parvovirus H-1PV and gemcitabine in pancreatic cancer. J Virol.

[31] Pei Q, Pan J, Ding X, Wang J, Zou X, Lv Y (2015). Gemcitabine sensitizes pancreatic cancer cells to the CTLs antitumor response induced by BCG-stimulated dendritic cells via a Fas-dependent pathway. Pancreatology.

[32] Le HK, Graham L, Cha E, Morales JK, Manjili MH, Bear HD (2009). Gemcitabine directly inhibits myeloid derived suppressor cells in BALB/c mice bearing 4 T1 mammary carcinoma and augments expansion of T cells from tumor-bearing mice. Int Immunopharmacol.

[33] Affara NI, Ruffell B, Medler TR, Gunderson AJ, Johansson M, Bornstein S (2014). B cells regulate macrophage phenotype and response to chemotherapy in squamous carcinomas. Cancer Cell.

[34] Karagiannis P, Gilbert AE, Josephs DH, Ali N, Dodev T, Saul L (2013). IgG4 subclass antibodies impair antitumor immunity in melanoma. J Clin Invest.

[35] Nanda S (2011). Cancer: CD40 agonists--a promising new treatment for pancreatic cancer?. Nat Rev Gastroenterol Hepatol.

[36] Hou YC, Chao YJ, Tung HL, Wang HC, Shan YS (2014). Coexpression of CD44-positive/CD133-positive cancer stem cells and CD204-positive tumor-associated macrophages is a predictor of survival in pancreatic ductal adenocarcinoma. Cancer.

[37] Di Caro G, Cortese N, Castino GF, Grizzi F, Gavazzi F, Ridolfi C, et al. Dual prognostic significance of tumour-associated macrophages in human pancreatic adenocarcinoma treated or untreated with chemotherapy. Gut. 2015. [Epub ahead of print]. 10.1136/gutjnl-2015-309193 26156960

[38] Sundararaj KP, Samuvel DJ, Li Y, Nareika A, Slate EH, Sanders JJ (2008). Simvastatin suppresses LPS-induced MMP-1 expression in U937 mononuclear cells by inhibiting protein isoprenylation-mediated ERK activation. J Leukoc Biol.

[39] Wang XQ, Luo NS, Salah ZQ, Lin YQ, Gu MN, Chen YX (2014). Atorvastatin Attenuates TNF-alpha Production via Heme Oxygenase-1 Pathway in LPS-stimulated RAW264.7 Macrophages. Biomed Environ Sci.

[40] Zhang O, Zhang J (2015). Atorvastatin promotes human monocyte differentiation toward alternative M2 macrophages through p38 mitogen-activated protein kinase-dependent peroxisome proliferator-activated receptor gamma activation. Int Immunopharmacol.

[41] Bradley MC, Hughes CM, Cantwell MM, Murray LJ (2010). Statins and pancreatic cancer risk: a nested case–control study. Cancer Causes Control.

[42] Carey FJ, Little MW, Pugh TF, Ndokera R, Ing H, Clark A (2013). The differential effects of statins on the risk of developing pancreatic cancer: a case–control study in two centres in the United Kingdom. Dig Dis Sci.

[43] Vinogradova Y, Coupland C, Hippisley-Cox J (2011). Exposure to statins and risk of common cancers: a series of nested case–control studies. BMC Cancer.

[44] Friedman GD, Flick ED, Udaltsova N, Chan J, Quesenberry CP, Habel LA (2008). Screening statins for possible carcinogenic risk: up to 9 years of follow-up of 361,859 recipients. Pharmacoepidemiol Drug Saf.

[45] Pastore A, Palleschi G, Fuschi A, Silvestri L, Al Salhi Y, Costantini E (2015). Can daily intake of aspirin and/or statins influence the behavior of non-muscle invasive bladder cancer? A retrospective study on a cohort of patients undergoing transurethral bladder resection. BMC Cancer.

[46] Ravnskov U, McCully KS, Rosch PJ (2012). The statin-low cholesterol-cancer conundrum. QJM.

