# Supplementary material for: Atorvastatin (Lipitor) attenuates the effects of aspirin on pancreatic cancerogenesis and the chemotherapeutic efficacy of gemcitabine on pancreatic cancer by promoting M2 polarized tumor associated macrophages
Source: J Exp Clin Cancer Res. 2016 Feb 16;35:33. doi: 10.1186/s13046-016-0304-4 (PMC4754966; doi:10.1186/s13046-016-0304-4)
Supplement: Additional file 1: Figure S1. — The illustration of the procedures of DMBA-induced pancreatic cancerogenesis model. Table S1. The list of antibodies. Table S2. The list of the primers of the real time RT-PCR. (DOCX 2437 kb) [file 13046_2016_304_MOESM1_ESM.docx]

Additional file 1


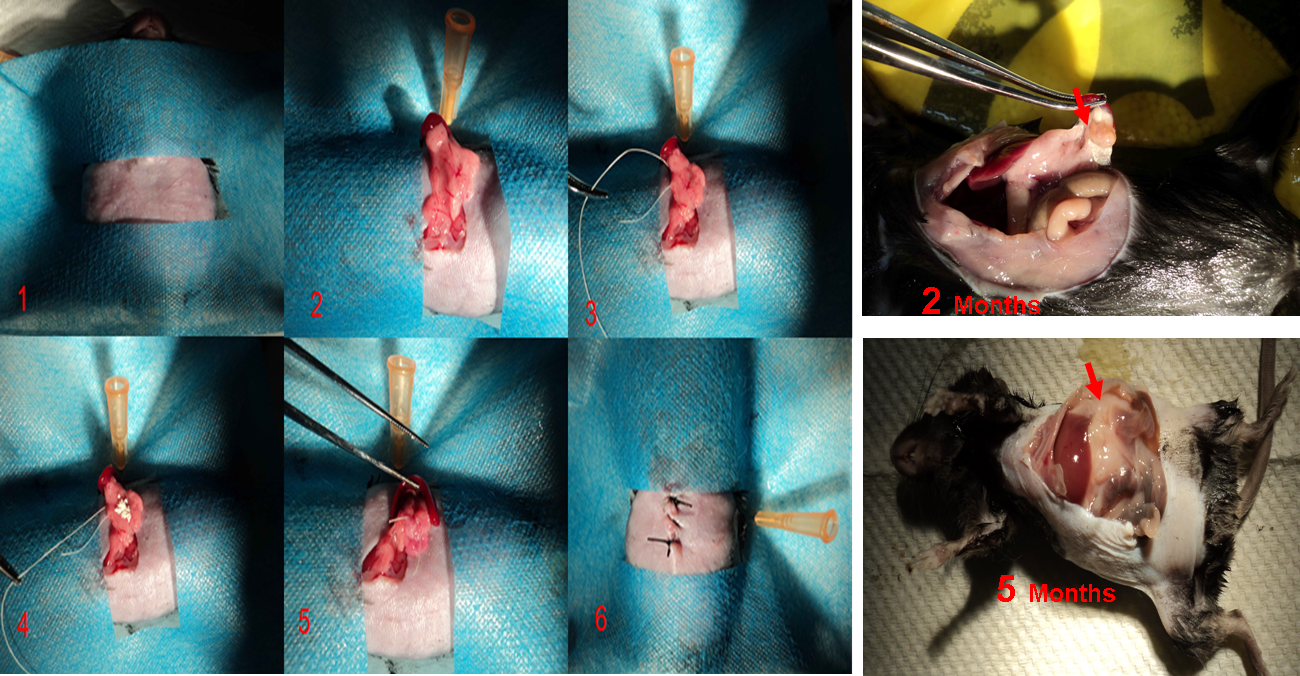


Figure.1 The illustration of the procedures of DMBA-induced pancreatic cancerogenesis model.1) Depilation using 10% Na_2_S solution, sterilization and draping the surgical region; 2) Median laparotomy; 3) Purse suturing; 4) 1mg DMBA implantation; 5) Knotting the purse suturing line; 6) Incision closing. Upper Right: A small mass occurred in the pancreas, 2 months after DMBA implantation (red arrow); Lower Right: A large mass occurred in the left upper abdomen, 5 moths after DMBA implantation (red arrow). Both of the masses were pathologically verified to be invasive pancreatic cancer.

Table.1 The list of antibodies

| Labelling | Antibody | Company | NO. |
| --- | --- | --- | --- |
| APC | CD45.2 | Biolegend | 109814 |
| Percp | CD45.2 | Biolegend | 109826 |
| Percp.cy5.5 | CD3 | BD | 560527 |
| FITC | CD4 | Biolegend | 100510 |
| PE | CD8a | Biolegend | 100708 |
| APC | CD19 | Biolegend | 115512 |
| FITC | CD19 | BD | 553785 |
| APC | CD11b | BD | 557657 |
| APC | CD11b | Biolegend | 101212 |
| PE | Gr-1 | BD | 553128 |
| FITC | F4/80 | Biolegend | 123108 |
| PE | CD16/32 | Biolegend | 101307 |
| APC | CD206 | Biolegend | 141708 |
| APC | CD11c | Biolegend | 117310 |
| FITC | NK1.1 | Biolegend | 108706 |
| PE | NKp46 | Biolegend | 137604 |
|  | Arginase-1 | Santa Cruz | SC-18351 |
|  | CD206 | Santa Cruz | SC-376108 |
| FITC | Rat IgG2b, κ Isotype Control | BD | 562308 |
| PE | Rat IgG2b, κ Isotype Control | BD | 556923 |
| Percp | Rat IgG2b, κ Isotype Control | BD | 550764 |
| APC | Rat IgG2b, κ Isotype Control | BD | 556924 |

Table 2 The list of the primers of the real time RT-PCR

| Gene | Sequence |
| --- | --- |
| mouse INF-γ up | GCGTCATTGAATCACACCTG |
| mouse INF-γ down | TGAGCTCATTGAATGCTTGG |
| mouse IL-2 up | CGGCATGTTCTGGATTTGAC |
| mouse IL-2 down | TCCACCACAGTTGCTGACTC |
| mouse IL-10 up | GGAGCAGGTGAAGAGTGATTTT |
| mouse IL-10 down | GGTACAAACGAGGTTTTCCAAG |
| mouse TGF-β up | GAAGGACCTGGGTTGGAAGT |
| mouse TGF-β down | TGGTTGTAGAGGGCAAGGAC |
| mouse IL-6 up | CTGCAAGAGACTTCCATCCAG |
| mouse IL-6 down | AGTGGTATAGACAGGTCTGTTGG |
| mouse TNF-α up | GAACTGGCAGAAGAGGCACT |
| mouse TNF-α down | GGTCTGGGCCATAGAACTGA |
| mouse Arg-1 up | CTCCAAGCCAAAGTCCTTAGAG |
| mouse Arg-1 down | AGGAGCTGTCATTAGGGACATC |
| mouse GAPDH up | GGCATTGCTCTCAATGACAA |
| mouse GAPDH down | TGTGAGGGAGATGCTCAGTG |
| mouse IL-4 up | ATCATCGGCATTTTGAACGAGG |
| mouse IL-4 down | TGCAGCTCCATGAGAACACTA |
